# Polymorphism of mycotoxin biosynthetic genes among *Fusarium equiseti* isolates from Italy and Poland

**DOI:** 10.1007/s13353-012-0085-1

**Published:** 2012-02-22

**Authors:** Łukasz Stępień, Karolina Gromadzka, Jerzy Chełkowski

**Affiliations:** 1 Department of Metabolomics, Institute of Plant Genetics, Polish Academy of Sciences, Strzeszyńska 34, 60-479 Poznań, Poland; 2 Department of Chemistry, Poznań University of Life Sciences, Wojska Polskiego 75, 60-625 Poznań, Poland

**Keywords:** Biosynthetic genes, DNA polymorphism, *Fusarium*, Mycotoxins, Zearalenone, Phylogenetic markers

## Abstract

*Fusarium equiseti* (Corda) Saccardo is a soil saprophyte and a weak pathogen, associated with several diseases of fruit and other crops in subtropical and tropical areas, but also in countries with temperate climate. A wide range of secondary metabolites has been identified among natural *F. equiseti* populations, with zearalenone (ZEA), fusarochromanone and fusarenon-X being the most common. In present study, the genetic diversity of strains from two populations (from Italy and Poland) was evaluated by analysing the translation elongation factor 1α (*tef*-1α) sequences, two polyketide synthases from the ZEA biosynthetic pathway (*PKS13* and *PKS4*) and the *TRI5* gene from the trichothecene biosynthetic pathway. ZEA was produced in rice cultures by 20 of the 27 tested isolates in concentrations ranging from 1.34 ng/g to 34,000 ng/g). The ability to produce enniatins and trichothecenes was evaluated in all strains by identifying *esyn1*, *TRI13* and *TRI4* genes. The presence of *PKS4* and *PKS13* genes was confirmed by polymerase chain reaction (PCR) in only some ZEA-producing isolates. Similarly, the *TRI5* gene was found in 14 of the 27 isolates tested. This is likely to have been caused by the divergence of those genes between *F. equiseti* and *F. graminearum* (the latter species was used for the primers design) and can be exploited in phylogenetic studies. The analysis of the mycotoxin biosynthetic gene sequences can be used to differentiate the studied genotypes even more precisely than the analysis of the non-coding regions (like *tef*-1α).

## Introduction

*Fusarium equiseti* (Corda) Saccardo is a common soil saprophyte associated with several fruit and other plant tissue diseases of agriculturally important crops. The fungus is typical mostly for subtropical and tropical areas, but is also isolated from soil and plant hosts in countries of temperate climate zone, like Norway, Canada, Russia and Poland (Booth 1971; Kosiak et al. 2005; Marasas et al. 1984). The list of host species includes cereals (wheat, triticale, maize and rice), asparagus, cotton, potato, tomato, cowpea, onion and ginseng (Punja et al. 2008). Besides cultivated plants, it also colonises wild species, including herbal plants and trees, e.g. *Equisetum* L., *Chenopodium* L. and *Pinus* L. Although *F. equiseti* is considered as moderately aggressive, it is capable of synthesising a vast range of toxic secondary metabolites, which exhibit both phyto- and cytotoxicity (Langseth et al. 1998–1999; Morrison et al. 2002). These include the trichothecenes: 4-acetylnivalenol (fusarenon X, FUS-X), nivalenol (NIV), scirpentriol with its acetyl derivatives (MAS and DAS), and numerous other mycotoxins, such as zearalenone (ZEA), beauvericin (BEA), fusarochromanone (FUSCHR), equisetine (EQ) and butenolide (Thrane 1989).

Recently, ZEA became particularly interesting to the researchers because of its oestrogenic properties and the ability to be accumulated at high levels in naturally infected cereal grain (Gromadzka et al. 2008). Besides *F. equiseti*, ZEA is produced by other *Fusarium* species, including the most important pathogens of maize and wheat, i.e. *F. culmorum* and *F. graminearum*. Also, glycosylated derivatives of ZEA were identified both in wheat and in maize grain. They can be regarded as potentially hazardous for human and animal health, as they can be converted to ZEA in the digestive tract (Berthiller et al. 2009; Sulyok et al. 2006). It can be accumulated in cereal grain at high levels, reaching several μg/g, and transferred with infected grain to cereal products, such as animal feed (Gromadzka et al. 2008).

Isolates of *F. equiseti* species are highly variable in a number of morphological traits, including the size of apical cells, conidia shape, aerial mycelium texture, growth rate on standard media, pigmentation and sporodochia appearance (Kosiak et al. 2005). Also, a great diversity has been observed in metabolites composition and amounts among natural *F. equiseti* populations, but ZEA, FUSCHR and FUS-X are the most commonly synthesised.

To identify and assess the genetic diversity of the natural populations of pathogens, numerous molecular assays have been developed and applied. As far as the *Fusarium* genus is concerned, analysis of the highly polymorphic intron regions like the translation elongation factor 1α (*tef*-1α) appears as the most useful and versatile tool, as it allows the discrimination of the genotypes even on the sub-specific level (Kristensen et al. 2005; Stępień et al. 2011a).

Therefore, a similar approach was used in this study in order to evaluate the variability of *F. equiseti* isolates from two populations (originating from Italy and Poland, plus an additional strain from Argentina), based on the partial sequences of the *tef*-1α. Additionally, we analysed the polymorphism present in the coding regions of the two genes from the ZEA biosynthetic pathway (*PKS13* and *PKS4*) and inside the *TRI5* gene, which encodes a key enzyme of the trichothecene biosynthesis pathway—trichodiene synthase. We have examined the level of ZEA biosynthesis in rice cultures and the ability of all isolates to produce enniatins/beauvericin and trichothecenes (from A and B groups) by the identification of the essential metabolic pathway genes *esyn1*, *TRI13* and *TRI4* using gene- and allele-specific polymerase chain reaction (PCR) assays.

## Materials and methods

### *Fusarium* strains and media

Twenty-seven *Fusarium* strains were included in the study. Twenty-five of them represented two distinct populations of *F. equiseti* (originating from Italy and Poland). One strain originating from Argentina (ITEM 4323) was also analysed as a representative genotype from a distinct population. Additionally, one *F. langsethiae* strain (KF 2640) was included in the study (Table 1). For the sequence analyses of *TRI5* and *PKS13* genes diversity, two strains of *F. graminearum* (KF 371 representing the nivalenol chemotype and KF 1413 representing the deoxynivalenol chemotype) were used as the outgroup. All isolates were identified using dedicated species-specific PCR-based markers (Table 2). Also, partial sequences of the *tef*-1α were amplified for molecular species identification and phylogenetic analysis. The providers of all strains were two culture collections: the KF *Fusarium* strain collection at the Institute of Plant Genetics, Polish Academy of Sciences, Poznań, Poland, and the ITEM collection at the Institute of Sciences of Food Production (ISPA-CNR), Bari, Italy.

**Table 1:** *Fusarium equiseti* isolates used in this study, along with their country of origin, presence of species-specific and *TRI5* gene-specific markers, as well as the amplicon sizes (in base pairs) of partial coding sequences of *PKS4* and *PKS13* genes from the zearalenone (ZEA) biosynthetic cluster

Isolate	Host	Origin	FeF/R	*TRI5*	*TRI4*	PKS4*	PKS13*
ITEM 4323	*Equisetum arvense*	Argentina	+	−	−	−	−
ITEM 2753 *F. avenaceum*	*Allium cepa*	Italy	−	−	−	500	−
ITEM 3190	*Chenopodium*	Italy	+	−	−	−	−
ITEM 3673	*Oryza sativa*	Italy	+	−	−	300	−
ITEM 3699	*O. sativa*	Italy	−	+	−	−	−
ITEM 4586	*Pinus* root	Italy	+	−	−	−	−
ITEM 4743	*Solanum lycopersicum*	Italy	+	−	−	−	−
ITEM 5104	*Orobanche ramosa*	Italy	+	−	−	750	−
ITEM 6164	*O. ramosa*	Italy	−	+	−	−	700
ITEM 6461	*Triticum* sp.	Italy	+	−	−	−	−
ITEM 6463	*Triticum* sp.	Italy	+	−	−	400	532
ITEM 7502	*Triticum* sp.	Italy	+	−	−	−	−
KF 2650	Soil	Italy	−	+	−	200/355	532
KF 2651	Soil	Italy	−	+	+	355	532
KF 8	Triticale	Poland	−	+	−	355	532
KF 13	*T. aestivum*	Poland	+	−	+	750	−
KF 24	*Zea mays*	Poland	+	+	−	750	532
KF 72	Triticale	Poland	−	+	+	355	532
KF 89	*T. aestivum*	Poland	−	+	−	355	532
KF 1011	*Lycopersicon esculentum*	Poland	+	+	−	750	532
KF 1017	*L. esculentum*	Poland	−	−	−	750	532
KF 2652	Soil	Poland	+	+	−	200/700	532
KF 2653	Soil	Poland	+	−	−	355/750	200/532
KF 2654	Soil	Poland	+	+	−	150/750	532
KF 2655	Soil	Poland	+	+	−	200/750	532
KF 2656	Soil	Poland	−	+	−	355	532
KF 2640 *F. langsethiae*	*T. aestivum*	Poland	−	+	+	−	700

*In base pairs

**Table 2:** Primers used in this study, expected amplicon sizes, references and GenBank accession numbers of the sequences amplified

Marker	5′ > 3′ sequence	Amplicon size (bp)	Accession number/reference
FeF1	CATACCTATACGTTGCCTCG	389	Mishra et al. 2003
FeR1	TTACCAGTAACGAGGTGTATG		
FaF	AGCATTGTCGCCACTCTC	920	Doohan et al. 1998
FaR	GTTTGGCTCTACCGGGACTG		
Flang	CAAAGTTCAGGGCGAAAACT	310	Wilson et al. 2004
Lanspo	TACAAGAAGAGCGTGGCGATAT		
Ef728M	CATCGAGAAGTTCGAGAAGG	600	Multiple
Tef1R	GCCATCCTTGGAGATACCAGC		
PKS4_F	AGACGGCGCAACAAGGGCTG	355	AY495638.1
PKS4_R	GCAGTTGCCCGTGTCGGACA		
PKS13_1	CCCAGCCAAGCCCAGTACGC	532	DQ019316.1
PKS13_2	ACAGCGGCTGACCTGGGTCA		
TRI4F1506	CCCCTGGCTACTCTCGAGA	550	Nicholson et al. 2004
T4EndR2	AAGCTTTGAGAACCTTCAC		
TRI5_1	AGCGACTACAGGCTTCCCTC	545	EF661664.1
TRI5_2	AAACCATCCAGTTCTCCATCT		
TRI13DON-F	CATCATGAGACTTGTKRAGTTTGGG	216	Nicholson et al. 2004
TRI13DON-R	GCTAGATCGATTGTTGCATTGAG		
TRI13NIV-F	CCAAATCCGAAAACCGCAG	308	Nicholson et al. 2004
TRI13NIV-R	TTGAAAGCTCCAATGTCGTG		
Esyn_1	GCCGTTGGCGAGCTGGTCAT	997	Z18755.3
Esyn_2	GCAAAGCACGCGTCAACGCA		

### Growth rates

The strains were examined for growth speed by plating on potato dextrose agar (PDA) medium. The growth speed of 25 isolates described as *F. equiseti* was measured as the colony diameter on 90-mm Petri plates with solid PDA medium (Oxoid, Basingstoke, Hampshire, UK) after 4 days from inoculation. The cultures were grown in the incubator set at room temperature (20°C) with a 12-h photoperiod. The average was calculated from two replicates.

### DNA extraction, PCR primers, cycling profiles and DNA sequencing

Genomic DNA of all isolates was extracted using a hexadecyltrimethylammonium bromide (CTAB) method described in detail previously (Stępień et al. 2008). All primers used for the PCR-based identification of mycotoxin biosynthetic genes, along with references or accession numbers of sequences used for their design, are presented in Table 2. PCRs were done in 25-μl aliquots using PTC-200 and C-1000 thermal cyclers (Bio-Rad, Hercules, CA, USA). Each reaction tube contained 1 unit of Platinum HotStart Taq DNA polymerase (Invitrogen, Carlsbad, CA, USA), 2.5 μl of 10× PCR buffer, 12.5 pmol of forward/reverse primers, 2.5 mM of each dNTP and about 10–20 ng of fungal DNA. The PCR conditions were as follows: 15 min at 95°C, 35 cycles of (30–60 s at 94°C, 30–60 s at 58-63°C, 1–2 min at 72°C) and 10 min at 72°C. Amplicons were electrophoresed in 1.5% agarose gels (Invitrogen) with ethidium bromide.

For sequence analysis, PCR-amplified DNA fragments were purified with exonuclease I (Epicentre, Madison, WI, USA) and shrimp alkaline phosphatase (Promega, Madison, WI, USA) using the following programme: 30 min at 37°C and 15 min at 80°C. Both strands were labelled using the BigDye Terminator 3.1 kit (Applied Biosystems, Foster City, CA, USA) according to Błaszczyk et al. (2005) and precipitated with ethanol. Sequence reading was performed using Applied Biosystems equipment.

### Sequence analysis and phylogeny reconstruction

The sequences of the PCR products were compared to the National Center for Biotechnology Information (NCBI) nucleotide collection using MEGABLAST and initially aligned with the ClustalW algorithm. Phylogenetic relationships were reconstructed with the MEGA4 software package (Tamura et al. 2007) using the maximum parsimony approach (closest neighbour interchange heuristics). No gap-containing positions were considered in the phylogeny analysis. All reconstructions were tested by bootstrapping with 10,000 replicates.

### Zearalenone analysis

Rice cultures were prepared from individual *Fusarium* isolates (Kostecki et al. 1999; Moretti et al. 2008). Long-grain white rice was used (50 g per flask with the addition of 12.5 ml of sterile water), left overnight and sterilised by autoclaving the next day. Samples were inoculated with 4 cm^2^ of 7-day-old mycelium on PDA medium. The average culture humidity was kept at around 30% and maintained for 14 days. Then, the cultures were dried at room temperature, ground and homogenised for 3 min with methanol–water (3:1, v/v) mixture (10 ml for 5 g of rice culture) and, finally, filtered through Whatman no. 4 filter paper (Sydenham et al. 1990).

ZEA content was determined by the high-performance liquid chromatography (HPLC) method according to the method of Visconti and Pascale (1998). The chromatographic system consisted of Waters HPLC 2695 apparatus with a Waters 2475 Multi λ Fluorescence Detector and a Waters 2996 Array Detector. The excitation and emission wavelengths were 274 and 440 nm, respectively. The reserved-phase C-18 Nova Pak column (3.9 × 150 mm) and acetonitrile–water–methanol (46:46:8, v/v/v) as the mobile phase at a flow rate of 0.5 ml/min were used. The detection limit of ZEA was 3 ng/g.

## Results

### Species-specific DNA marker detection, *tef*-1α sequence analysis and phylogeny reconstruction

**Fig. 1:**
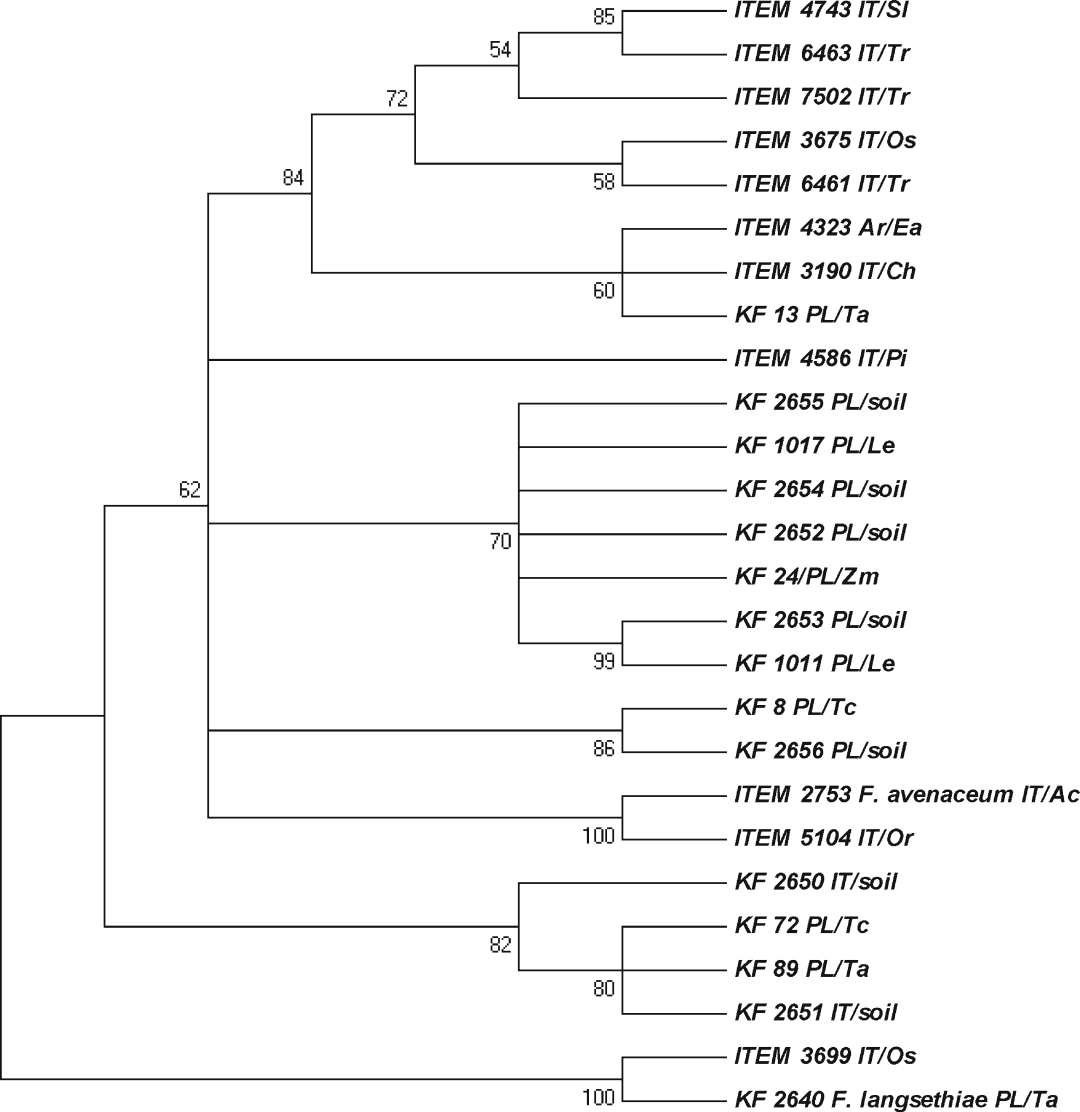
Most parsimonious tree for the isolates studied, created on the basis of the translation elongation factor 1α (*tef*-1α) sequences. The tree was obtained using the maximum parsimony approach and tested by bootstrapping (10,000 replicates) with a cut-off value of 50%. Abbreviations used for the origin and host species: IT – Italy, PL – Poland, AR – Argentina, Os – *Oryza sativa*, Zm – *Zea mays*, Ea – *Equisetum arvense*, Ac – *Allium cepa*, Ch – *Chenopodium*, Pi – *Pinus* root, Sl – *Solanum lycopersicum*, Or – *Orobanche ramosa*, Tr – *Triticum* sp., Le – *Lycopersicon esculentum*, Tc – triticale, Ta – *Triticum aestivum*

Three species-specific DNA markers FeF1/R1, FaF/R and Flang/Lanspo (Table 2) were used to identify the strains included in the study. Of the 25 *F. equiseti* strains studied, 16 displayed the amplicon of expected size, while nine genotypes did not amplify the fragment (Table 1). In the cases where, based on the molecular data from species-specific marker detection, we were not able to confirm the species identification, we still could prove it thanks to the sequence similarities to the *tef*-1α sequences of *F. equiseti* available in the GenBank sequence database using BLASTn. The primers Ef728M/Tef-1R were used successfully in many fungal species and they worked fine in our case, amplifying the fragment of *tef*-1α gene using template from all isolates except one (ITEM 6164). Among 27 isolates studied, 22 presented the highest similarity to GenBank accessions of *F. equiseti* (90–99%). The isolate ITEM 2753 appeared to be *F. avenaceum* and KF 2640 was confirmed as *F. langsethiae*. The ITEM 3699 strain was the most similar to *F. langsethiae* and the ITEM 5104 strain to *F. avenaceum* (Fig. 1). To investigate the strain ITEM 6164 in more detail, a *tef*-1α gene fragment adjacent to the standard one was amplified and sequenced using an alternative pair of primers. It was dedicated to identifying other fungal genera, but also with some *Fusarium* genotypes (results not shown). It displayed the highest level of similarity to *F. sporotrichioides* (97%), *F. graminearum* (97%) and *F. fujikuroi* (97%). All the obtained *tef*-1α sequences (except ITEM 6164) were submitted to the NCBI GenBank and are publicly available under accession numbers JF966231–JF966256.

### Growth rate

**Fig. 2:**
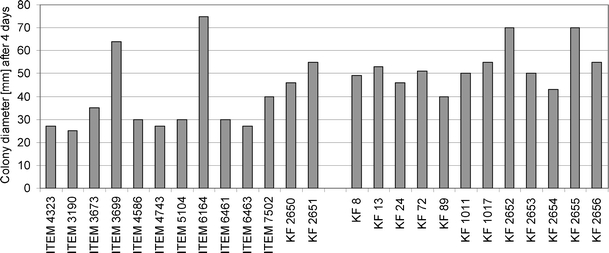
Colony diameter of the strains used in the study after four days of culturing on potato dextrose agar (PDA) medium at room temperature (mean of two replicates)

Petri plates with PDA medium were used for the analysis of the growth speed of 25 *F. equiseti* strains used in the study. In the group of Polish strains, only two genotypes measured 70 mm (KF 2652 and KF 2655), while all the remaining isolates ranged from 40 to 55 mm (Fig. 2). The majority of Italian strains grew more slowly than the Polish group, forming a colony of about 25–30 mm in diameter after four days. However, some exceptions from this rule were recorded (ITEM 3699 and ITEM 6164 reached 64 and 75 mm, respectively). Also, two isolates originating from Italy (KF 2650 and KF 2651) presented growth speed on the level of Polish strains rather than of the Italian group (Fig. 2).

### Quantification of zearalenone

**Fig. 3:**
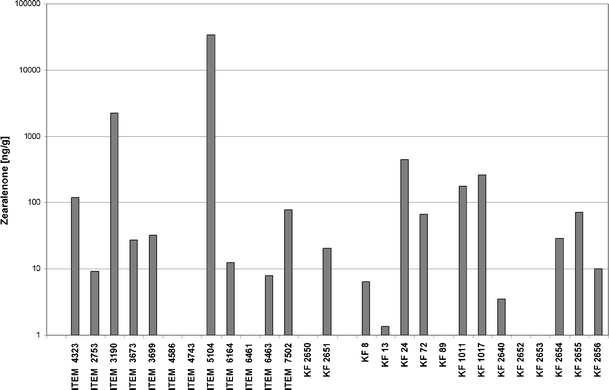
Zearalenone (ZEA) production by *Fusarium equiseti* isolates used in this study

ZEA biosynthesis levels varied among all the *F. equiseti* strains analysed (Fig. 3). ZEA was detected in 20 of the 27 tested isolates. ZEA was produced by *F. equiseti* in concentrations ranging between 1.34 and 34,237 ng/g (>34 μg/g). The highest toxin content was observed in Italian isolates: ITEM 5104 and ITEM 3190 (34,237 and 2273.2 ng/g respectively). The other *Fusarium* isolates were characterised by much lower abilities of the mycotoxin biosynthesis: 14 isolates produced less than 100 ng/g of the toxin and four isolates’ ZEA content only reached the range of 120–460 ng/g.

### Sequence polymorphism in two genes from the zearalenone biosynthetic pathway

**Fig. 4:**
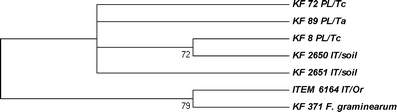
Most parsimonious tree for six chosen isolates based on the sequences of the polyketide synthase (PKS13) gene from the ZEA metabolic pathway. The tree was obtained using the maximum parsimony approach and tested by bootstrapping (10,000 replicates) with a cut-off value of 70%. The *F. graminearum* KF 371 strain was used as the reference. Abbreviations used for the origin and host species: IT – Italy, PL – Poland, Or – *Orobanche ramosa*, Tc – triticale, Ta – *Triticum aestivum*

Based on the sequences of two *F. graminearum* genes encoding polyketide synthases involved in ZEA biosynthesis (*PKS4* and *PKS13*), we designed PCR primers to amplify portions of those genes using genomic DNAs from *F. equiseti* strains. Eight strains from the Italian population of *F. equiseti* did not yield any amplification using the *PKS4*-specific marker, while among the remaining strains (including *F. avenaceum* ITEM 2753), eight different amplicons were identified (Table 1). Concerning the *PKS13* locus, most of the strains displayed the 532-bp allele and two (*F. langsethiae* KF 2640 and ITEM 6164) amplified a fragment of about 700 bp in length. Eleven strains gave no amplification at all (almost all were from the Italian group). Thus, the *PKS4* gene seems to be more polymorphic than *PKS13*, especially among the isolates from the Polish population. Sequence analysis of the amplified fragments should be performed to reveal the level of this polymorphism, as some of the amplicons can be non-specific. We sequenced several chosen fragments of the *PKS13* sequence to confirm the specificity of designed markers and to analyse their intra-specific diversity, as well as similarities between *F. equiseti* and *F. graminearum*. Based on the aligned sequences, a dendrogram was designed (Fig. 4). All the obtained *PKS13* sequences were submitted to the NCBI GenBank and are publicly available under accession numbers JF966273–JF966279.

### Identification of enniatin and trichothecene biosynthetic genes

To further examine the toxigenic abilities of the strains studied, we used gene-specific DNA markers to prove or exclude the capability of the strains to produce enniatins (by identifying the *esyn1* gene) and trichothecenes (by identifying the *TRI5* gene and two alleles of the *TRI13* gene, associated with deoxynivalenol and nivalenol chemotypes). In case of the *esyn1* gene, encoding enniatin synthetase, we could identify it only in the strain of *F. avenaceum* ITEM 2753. Also, we did not find any amplification products from the strains of *F. equiseti* using primers specific for TRI13DON and TRI13NIV markers, which suggests the inability of those strains to produce DON or NIV. *F. graminearum* strains KF 1413 and KF 371, exhibiting DON and NIV chemotypes, respectively, amplified the expected amplicons (results not shown).

**Fig. 5:**
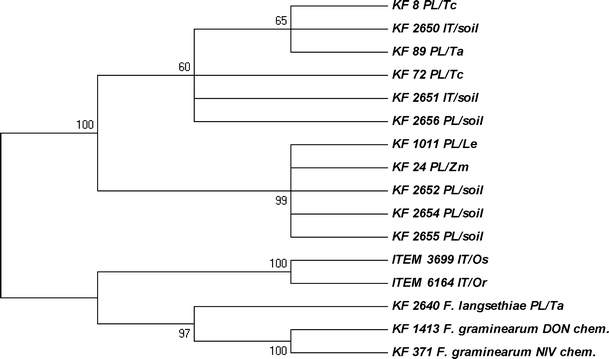
Most parsimonious tree for the 14 strains tested, based on the trichodiene synthase (*TRI5*) gene sequences. The maximum parsimony approach was used and tested by bootstrapping (10,000 replicates) with a cut-off value of 50%. Two strains of *F. graminearum* (DON chemotype, KF 1413, and NIV chemotype, KF 371) were used as references. Abbreviations used for origin and host species: IT – Italy, PL – Poland, Os – *Oryza sativa*, Zm – *Zea mays*, Or – *Orobanche ramosa*, Le – *Lycopersicon esculentum*, Tc – triticale, Ta – *Triticum aestivum*

Since *F. equiseti* has often been reported as a group A trichothecene producer, we screened the studied strain set for the presence of the *TRI5* gene and an allele of the *TRI4* gene specific for A-trichothecene producers (Nicholson et al. 2004). Only 14 of the strains possessed the *TRI5* gene: 13 *F. equiseti* genotypes and KF 2640 *F. langsethiae* strain (Table 1). The amplified fragments were used to read the sequences, which served as the basis for phylogenetic tree construction (Fig. 5). All the obtained *TRI5* sequences were submitted to the NCBI GenBank and are publicly available under accession numbers JF966257–JF966272.

## Discussion

The results of the species-specific DNA marker identification showed that the set of *F. equiseti* isolates used in the study reveals a high level of intra-specific polymorphism. Nine of 25 isolates tested did not amplify the expected amplicon (Table 1). There was no correlation between the country of origin and the presence of the marker fragment. The marker was designed on the basis of the highly variable ITS region of nuclear ribosomal DNA (Mishra et al. 2003), so it seems likely that it displays intra-specific polymorphism, which can explain our lack of amplification and identification problems. However, after analysing the *tef*-1α sequence, it was proven that eight of nine isolates still represent *F. equiseti*, despite the lack of the amplification of the FeF/FeR marker. Similarly, the analysis of *tef*-1α has proven a considerably high level of polymorphism, as it was already postulated before for other members of the *Fusarium* genus (Kristensen et al. 2005; Stępień et al. 2011b).The only exception was ITEM 6164 strain. We only were able to amplify a fragment of the *tef*-1α gene using primers designed for the *Clonostachys* genus and amplifying a region adjacent to the studied one (results not shown). It is rather unusual concerning the considerable level of conservation present inside this gene (Geiser et al. 2004; Kristensen et al. 2005). The latest findings suggest that sequences which are unique at a species (or group of species) level (e.g. mycotoxin biosynthetic genes) serve as better targets for designing species-specific markers (Alexander et al. 2011; Fernández-Ortuño et al. 2010; Niessen et al. 2004; Proctor et al. 2009; Stępień et al. 2011a).

Based on the obtained *tef*-1α sequences, an alignment was prepared and the dendrogram of similarities was calculated (Fig. 1). Concerning only the country of origin, groups of isolates are dispersed, although a group of seven Polish strains from soil, *L. esculentum* and *Z. mays* is distinguishable: KF 2652, KF 2653, KF 2654, KF 2655, KF 24, KF 1011 and KF 1017. What is even more interesting, a group of four isolates (KF 72, KF 89, KF 2650 and KF 2651) is clearly sharing a rather uncommon variant of the gene, regardless of the origin (two are IT and the other two PL, see Fig. 1). A high level of diversity among *F. equiseti* strains is not surprising, as it was already shown that the *tef*-1α gene displays a considerable level of polymorphism among the genotypes of this species (Punja et al. 2008). Also, using different techniques, like restriction fragment length polymorphism (RFLP) and thin layer chromatography (TLC) of the synthesised metabolites, it was possible to differentiate the isolates of common geographical origin (Kosiak et al. 2005). Another remarkable observation is a similarity between the strain ITEM 2753, which was confirmed as *F. avenaceum*, and ITEM 5104. The most likely explanation for this is the misidentification of the strain. However, concerning morphological traits typical for *F. equiseti*, as well as the presence of the FeF/FeR and *PKS4* gene markers (Table 1) and the high level of the in vitro ZEA biosynthesis (Fig. 3), it is also possible that ITEM 5104 strain represents the “off-type” *F. equiseti* genotype.

As far as growth speed analysis is concerned, the majority of Polish *F. equiseti* strains grew slightly faster than the Italian ones (Fig. 2). There were two exceptions, however: the strains ITEM 3699 and ITEM 6164 were the fastest growing from the IT population. It seems likely that populations originating from Southern (i.e. warmer) locations will have an optimum temperature higher than those from temperate climate (PL population). Kosiak et al. (2005) recorded similar colony sizes (39–68 mm) for Norwegian isolates as those observed in this study for Polish ones. Canadian isolates from ginseng fields displayed the fastest growth at about 26°C (Punja et al. 2008), which seems to be in good agreement with our data. The ITEM 3699 strain was the only one that produced carmine reddish pigment in a PDA culture (results not shown). Having analysed the morphology of conidia and mycelium, and taking the *tef*-1α sequence into consideration, it seems likely that the strain represents *F. langsethiae* rather than *F. equiseti*. ITEM 6164 strain’s morphology is more typical to *F. equiseti*, but after the strain was subjected to further investigation by analysing the mycotoxin biosynthetic genes *TRI5* and *PKS13* (Figs. 4 and 5), it appeared that it probably represents *F. sporotrichioides*.

There are only a few publications on the biosynthesis of ZEA by *F. equiseti* because, until recently, the species was considered as being unable to synthesise ZEA, and its main metabolites were fusarochromanone and trichothecenes (Marasas et al. 1984; Morrison et al. 2002). According to Bosch and Mirocha (1992), ZEA was detected in 8 of the 23 tested isolates of *F. equiseti* at concentrations between 55 and 3,433 μg/g. Two of them were lethal to rats, although in culture extracts, the only toxin found was ZEA, at concentrations of 567 and 232 μg/g, respectively. According to Langseth et al. (1998–1999), who examined mycotoxin production by *F. equiseti* isolated from Norwegian cereals, three isolates produced ZEA in the range 0.1 to 3 μg/g. Kosiak et al. (2005) also found ZEA in all 25 examined isolates of *F. equiseti*, but only 14 of them synthesised this toxin in substantial amounts.

Concerning the relatively high level of ZEA biosynthesis by IT strains (Fig. 3), it seems unexpected that most of them did not amplify *PKS4* and *PKS13* genes (Table 1). On the contrary, Polish isolates, which generally produced less toxin, showed the presence of both genes, with only one exception (KF 13 did not amplify the *PKS13* gene). Obviously, all strains that produce ZEA are supposed to carry *PKS* genes, despite the negative PCR results. This inconsistency may be explained by the fact that the primers were designed on the basis of *F. graminearum* gene sequences, so a significant level of inter-specific divergence is possible. Specific markers for the reliable identification of *PKS* genes from *F. equiseti* will be created for future research on the basis of the sequences obtained. Interestingly, similar differentiation of both populations can be visible in the case of *TRI5* gene identification. Almost all Polish isolates displayed the presence of the gene, while the Italian isolates did not (Table 1). Again, in the case of the *PKS13* sequence, the ITEM 6164 strain appeared as being different from the other strains of *F. equiseti*, grouping with *F. graminearum* (Fig. 4) with a similarity level exceeding 90%.

The abilities of all strains to produce other toxins, such as enniatins and trichothecenes, were analysed by identifying the genes involved in two metabolic pathways. Those were: enniatin synthase gene *esyn1*, trichodiene synthase gene *TRI5*, together with *TRI4* marker specific for group A trichothecene producers and *TRI13*-based markers distinguishing deoxynivalenol and nivalenol chemotypes among trichothecene producers (Nicholson et al. 2004). The *esyn1* gene was present only in the ITEM 2753 strain of *F. avenaceum*, which is the species considered as one of the major producers of this mycotoxin group (Jestoi et al. 2004; Langseth et al. 1998–1999; Logrieco et al. 2002). However, since *F. equiseti* often produces beauvericin and both cyclohexadepsipeptides (beauvericin and enniatins) share a common biosynthetic pathway, there is a strong possibility that the species possess some form of the synthetase encoded by the *esyn1* gene homologue. Experimental work to prove its presence in *F. equiseti* using newly designed primers is currently being carried out. Also, both markers amplifying portions of the *TRI13* gene gave no amplification from all the strains tested, which should be regarded as the inability to produce DON or NIV by *F. equiseti*. Actually, the lack of DON/NIV chemotype markers suggests the ability to synthesise group A trichothecenes, like scirpentriol, MAS or DAS, which are, in fact, often produced by this species (Kosiak et al. 2005). *TRI4* marker was found in only three strains of *F. equiseti* and one *F. langsethiae* strain (Table 1). This is likely a result of inter-specific sequence divergence between *F. graminearum* and *F. equiseti*; however, the marker, despite being described as ”generic”, has not yet been tested on *F. equiseti* isolates to prove its reliability (Nicholson et al. 2004). Weak amplification fragments obtained in the present work will serve as templates to design species-specific *TRI4* markers.

We found the *TRI5* gene present in almost all strains from the Polish population and only in a few isolates originating from Italy (Table 1). Two of them (KF 2650 and 2651), though described as Italian strains, based on the *tef*-1α and *TRI5* sequence comparisons, are placed firmly inside the clade of Polish isolates (Figs. 1 and 5). Also, the speed of their growth resembles the PL strains rather than the IT ones. When *tef*-1α and *TRI5* dendrograms were compared, two distinct groups of PL isolates from the *tef*-1α tree seem to be more dispersed when the *TRI5* sequence is analysed. It can be concluded that the Polish strain set used in our study was less uniform than the Italian set, although more isolates should be added to the analyses we have presented here in order to support this hypothesis. Two other strains (ITEM 3699 and ITEM 6164) were already highlighted on the basis of the partly controversial results of the sequence analysis (Figs. 1 and 4). They formed a separate branch on the *TRI5* tree, being more closely related to *F. langsethiae* (KF 2640) and *F. graminearum* variants (KF 1413 and KF 371), as well as to the *F. sporotrichioides* sequences of *TRI5* present in GenBank (results not shown), than to those present in the isolates from the Polish population of *F. equiseti* (Fig. 5).

All our results provide quite a novel insight into the intra-specific polymorphisms of the mycotoxin biosynthetic gene sequences in *F. equiseti*. It shows the divergence of those genes between the species but considerably higher conservation level inside the species. A general conclusion can be drawn that the coding sequences of mycotoxin biosynthetic genes (like *TRI5*) are as good target sequences for species and population discrimination as highly variable intron regions of genes widely used in phylogenetic studies (e.g. *tef*-1α) and, in some cases, they even have some advantages over the less diverse ones, like the ITS regions.
